# Supplementary material for: Transcriptome Analyses of Diaphorina citri Midgut Responses to Candidatus Liberibacter Asiaticus Infection
Source: Insects. 2020 Mar 7;11(3):171. doi: 10.3390/insects11030171 (PMC7143376; doi:10.3390/insects11030171)
Supplement: Supplementary file 1 [file insects-11-00171-s001.zip › Supplementary File/Table S1.docx]

Table S1. All primers used for RT-qPCR in this study.

| **Gene ID** | **Best match** | **Primer sequences (5’ to 3’)** | **Length (bp)** |
| --- | --- | --- | --- |
| DcitrP055490.1 | E3 ubiquitin-protein ligase | CGGGAAATGAACCCCAATGA | 202 |
|  |  | CGCAGGTCTGACAGGAAGTAATG |  |
| DcitrP062105.1 | Proteasome activator complex subunit 3-like | GAAATGAAAATGAGAGCAAAGAGA | 110 |
|  |  | CGATGGAGTGAGAATCAGAGTGG |  |
| DcitrP026550.1 | Ubiquitin-associated domain-containing protein 1 | AAGCACCCTTGGATTTCAGTATG | 199 |
|  |  | TGTGGAGGTATTGGAGGTTTAGC |  |
| DcitrP080605.1 | Ubiquitin-conjugating enzyme E2 J2 | GTCATAGCAGAACCAAACCCAGC | 127 |
|  |  | TGAATGGGAAATCTCTTGGAAAA |  |
| DcitrP013835.1 | 40S ribosomal protein S9 | GTGAGAATTGGAGTGCTTGATGA | 207 |
|  |  | GCGTACTACGAAACTTGGGATGT |  |
| DcitrP037015.1 | 28S ribosomal protein S27, mitochondrial | GTCTTGGACTAACAAAGGATGCC | 155 |
|  |  | ACTCTCATTGCTACTGCTGGGTG |  |
| DcitrP036095.1 | Spondin-2-like | TACATTCTACGCTCCACCCATCA | 147 |
|  |  | CACAAATCCAAACTATCCACTCCA |  |
| DcitrP022220.1 | Apolipophorin-III | CCGAGACCGTCAAATCTATCCA | 221 |
|  |  | GACGACTTGCTTGCTCAACTTCT |  |
| DcitrP017415.1 | Heat shock protein 70 | TCATCACAGTCCCCGCCTATT | 184 |
|  |  | GTTCCGCCTCCCAAGTCAAA |  |
| DcitrP034560.1 | Partitioning defective 6 like protein gamma | TGGAACACCTGGGAAGACAAAA | 196 |
|  |  | ACCAGTAGAAGTGACACGCAGG |  |
| DcitrP079275.1 | Cytochrome P450 CYP4C4 | GCTGGTCTCTCTTCCTCCTGG | 110 |
|  |  | ATCTCGCATCGTGATTTTCCG |  |
| DcitrP045800.1 | Probable cytochrome P450 6a14 | TCTTATCAACTTCCCAACACCG | 217 |
|  |  | GCTGAGCGAATCTTTTACCAATG |  |
| DcitrP098450.1 | Acetylcholine receptor subunit alpha | CCTGGGTGTATGACGGGAAC | 182 |
|  |  | TCTGAGTCGGAAGTGAATGGTG |  |
| DcitrP034805.1 | Gelsolin | GTGACTGTCGCCTTATTTGGTG | 119 |
|  |  | AGGAACTCTCCCAGCATTTACAA |  |
|  | GAPDH | CATGGCAAGTTCAACGGTGA | 171 |
|  |  | CGATGCCTTCTCAATGGTGG |  |
